# Circular RNAs: Novel Regulators of Neuronal Development

**DOI:** 10.3389/fnmol.2016.00074

**Published:** 2016-08-26

**Authors:** Daniëlle van Rossum, Bert M. Verheijen, R. Jeroen Pasterkamp

**Affiliations:** ^1^Department of Translational Neuroscience, Brain Center Rudolf Magnus, University Medical Center UtrechtUtrecht, Netherlands; ^2^Department of Neurology and Neurosurgery, Brain Center Rudolf Magnus, University Medical Center UtrechtUtrecht, Netherlands

**Keywords:** circular RNA, non-coding RNA, microRNA, splicing, neuronal development, neurological disorders

## Abstract

Circular RNAs (circRNAs) are highly stable, circularized long non-coding RNAs. circRNAs are conserved across species and appear to be specifically enriched in the nervous system. Recent studies show that many circRNAs are expressed in a tissue- and developmental-stage-specific manner, reveal a striking regulation of circRNAs during neuronal development, and detect their presence at synaptic sites. The exact functions of circRNAs remain poorly understood, but evidence from analysis of some circRNA molecules suggests that they could substantially contribute to the regulation of gene expression, particularly in architecturally complex and polarized cells such as neurons. Emerging evidence also indicates that circRNAs are involved in the development and progression of various neurological disorders. In this review, we summarize the molecular characteristics of circRNAs and discuss their proposed functions and mechanism-of-action in developing neurons.

## Introduction

Circular RNAs (circRNAs) are a class of long non-coding RNAs (lncRNAs) that were originally described in the late 1970s. circRNAs were first detected in human cell lines using electron microscopy (Hsu and Coca-Prados, 1979), adding to a number of observations of circRNAs in other experimental models (Sanger et al., 1976; Arnberg et al., 1980). The serendipitous discovery of circular transcripts generated from the gene *deleted in colorectal cancer* (*DCC*), which encodes a cell surface receptor for the guidance cue Netrin-1, demonstrated that circRNAs can actually originate from transcribed genes (Nigro et al., 1991). In the following years, circRNA isoforms derived from several other loci were found in cells, including from the *ETS-1, Dystrophin* and *Cytochrome P-450 2C18* genes (Cocquerelle et al., 1992; Saad et al., 1992; Bailleul, 1996; Zaphiropoulos, 1997). Initially, these circRNAs were thought to be potentially pathogenic byproducts of aberrant splicing or ‘transcriptional/splicing noise’ and did not receive much attention. However, skepticism toward the existence and significance of circRNAs faded away with the development of new and improved technical approaches in transcriptomics and bioinformatics. High-throughput sequencing of ribosome-depleted RNA convincingly showed that human fibroblasts accommodate more than 25.000 unique and stable circRNAs (Jeck et al., 2013). These circRNAs originated from 14,4% of the expressed genes, which came as a surprise, as circRNAs were considered a rare phenomenon. Estimates indicate that circRNAs may actually account for about 1% of total RNA in human (Salzman et al., 2013).

Recently, a plethora of studies reported the expression of a variety of circRNAs in different species ranging from human and mouse to *Drosophila* and *C. elegans* (Salzman et al., 2012, 2013; Jeck et al., 2013; Memczak et al., 2013; Guo et al., 2014; Wang et al., 2014; Westholm et al., 2014; Zhang et al., 2014; Venø et al., 2015). These studies demonstrate that circRNAs are evolutionary conserved and expressed in a time-, cell type- and gene-specific manner. While most circRNAs are lowly abundant, some are ubiquitously expressed and are present at higher copy numbers (>10-fold) as compared to their linear transcripts (Jeck et al., 2013; Salzman et al., 2013). Interestingly, these studies also revealed that circRNAs are notably regulated during neuronal differentiation and nervous system development, while highlighting specific subcellular distributions of certain circRNAs in neurons, e.g., in the synapse (Rybak-Wolf et al., 2015; Venø et al., 2015; You et al., 2015). These findings hint at important roles for circRNAs in neural development and function. Here, we review the molecular characteristics of circRNAs and discuss their proposed functions and mechanism-of-action in developing neurons.

## Circrna Biogenesis

### Back-Splicing and Composition

**FIGURE 1:**
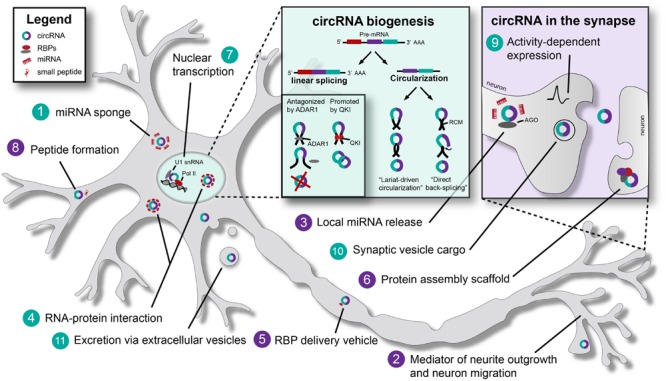
**Biogenesis and (putative) functions of Circular RNAs (circRNAs) in neurons.** Schematic representation of a neuron, indicating established (*turquoise*) and putative (*purple*) general and neuron-specific functions of circular RNAs (circRNAs): **(1)** circRNAs such as CDR1as (Cerebellar degeneration-related protein 1 antisense) and SRY (Sex determining region Y) have binding sites for specific miRNAs, e.g., miR-7. By binding these miRNAs they act as ‘miRNA sponges’ and inhibit miRNA function. **(2)** circRNAs may affect neurite outgrowth and neuronal migration. For example, ectopic CDR1as expression causes migration defects *in vitro*. **(3)** miR-671 can bind CDR1as to induce AGO-mediated cleavage of this circRNA. This may result in the local release of other miRNAs bound by CDR1as. **(4)** Various RNA-binding proteins (RBPs) bind circRNAs. For example, MBL (muscleblind) can bind to its own circRNA. QKI (Quaking) and ADAR1 (Adenosine deaminase acting on RNA 1) bind circRNAs to regulate circRNA biogenesis (see box titled *circRNA biogenesis*). circRNAs may ‘sponge’ RBPs (such as TDP-43) in the nucleus to regulate gene expression. **(5)** Binding of RBPs by circRNAs may facilitate RBP transport to distal compartments in neurons, e.g., synapses. **(6)** circRNAs have been proposed to act as scaffolds for protein complex assembly. By binding multiple RBPs, circRNAs might facilitate stable interactions between proteins. **(7)** Exon-intron circRNAs (EIciRNAs) are associated with RNA polymerase II (Pol II) in the nucleus. EIciRNAs recruit Pol II to regulate host gene transcription via U1 snRNA. **(8)** It has been proposed that small peptides and/or proteins may be generated from circRNAs in the cytoplasm. **(9)** The expression of certain circRNAs is affected by neuronal activity. **(10)** circRNAs are present in synaptic vesicles. **(11)** circRNAs are present in extracellular vesicles and could act as long range signaling molecules. RCM, reverse complementary matches; Pol II, RNA polymerase II; AGO, Argonaute.

Circular RNAs can be distinguished from their linear counterparts by their remarkable continuous closed loop structure, formed by a ‘*back-splicing*’ event wherein a covalent bond is formed between 5′ (splice donor) and 3′ (splice acceptor) splice-sites of a pre-mRNA (Jeck et al., 2013; Memczak et al., 2013). Back-splicing leads to the formation of a ‘head-to-tail junction’ that contains a unique sequence not present in mRNAs. Because they lack 5′ and 3′ termini, circRNAs do not show typical features of mRNA processing, such as 5′ capping and a poly(A)-tail, making them highly resistant to degradation by exonucleases. The biogenesis of circRNAs is poorly understood, but appears to be distinct from canonical pre-mRNA splicing that occurs during the maturation of mRNA transcripts. However, back-splicing reactions do seem to be dependent on canonical splicing machinery (Ashwal-Fluss et al., 2014; Starke et al., 2015). Different mechanisms such as ‘*direct back-splicing*’ or ‘*lariat-driven-circularization*’ have been proposed to explain the generation of circRNAs (Jeck et al., 2013; Memczak et al., 2013; Ashwal-Fluss et al., 2014) (**Figure 1**). circRNAs are very heterogeneous and can (1) consist of coding or scrambled coding sequences only, (2) contain exonic and intronic segments, i.e., in exon-intron circRNAs (EIciRNA) that retain flanking intronic sequences (Zhang et al., 2013; Li et al., 2015b), or (3) be derived from untranslated regions (UTRs), intergenic loci, and antisense sequences. It is evident that these structurally diverse circRNAs could fulfill pleiotropic functions. A detailed overview of mechanisms for circRNA formation is beyond the scope of this review, but readers are directed to other articles on this subject (Chen and Yang, 2015; Chen, 2016; Ebbesen et al., 2016).

### Regulation of CircRNA Biogenesis

Circular RNA biogenesis and expression likely depend on various *cis*-regulatory elements and *trans*-acting factors. Evidence for the regulation of circRNA formation in *cis* comes from the identification of inverted repeat sequences bracketing the region that produces circRNAs. Highly abundant circRNAs have significantly longer flanking introns, and genes giving rise to circRNA transcripts generally contain longer intronic sequences as compared to genes that do not (Salzman et al., 2012; Zhang et al., 2013). In line with this, circRNAs originating from genes with neuronal functions in *Drosophila* often have long introns (Westholm et al., 2014). Similarly, genes involved in axon guidance and Wnt signaling give rise to a disproportionally large number of circRNAs in pig brain and have, on average, long intronic sequences (Venø et al., 2015). These long intronic regions may act to slow down canonical splicing, allowing back-splicing to occur. In this way, circRNA formation competes with pre-mRNA splicing. Intronic elements flanking circRNA transcripts, such as ALU-repeats, have been demonstrated to be involved in the circularization of exons (Dubin et al., 1995; Salzman et al., 2012; Jeck et al., 2013; Ashwal-Fluss et al., 2014; Liang and Wilusz, 2014; Ivanov et al., 2015). Importantly, circRNA expression is not always correlated with expression of linear RNA from the same locus. Therefore, *cis* motifs alone are not sufficient to explain the dynamic expression patterns of circRNA.

*Trans-*acting factors that can affect circRNA biogenesis are RNA-binding proteins (RBPs), including splicing factors. RBPs carry out essential roles during neural development and their dysregulation contributes to neural disorders. Deficiency of the RBP Quaking (QKI) for instance may contribute to schizophrenia pathogenesis and the development of human inherited ataxia (Åberg et al., 2006; Mulholland et al., 2006). Interestingly, knockdown of QKI reduces the production of circRNAs, while integration of QKI binding sites into linear RNAs induces exon circularization. These observations indicate a role for QKI in the formation of circRNAs (Conn et al., 2015). In contrast to the agonistic effect of QKI, the RBP ADAR1 (Adenosine deaminase acting on RNA 1) inhibits the formation of circRNAs by binding double stranded RNA (**Figure 1**) (Chen and Carmichael, 2009; Wahlstedt et al., 2009; Osenberg et al., 2010; Shtrichman et al., 2012; Ivanov et al., 2015). It will be interesting to determine whether QKI’s and ADAR1’s role in neural development and disease are directly mediated through their ability to control circRNA biogenesis.

## Neuronal Functions of Circrnas

The functional role of most circRNAs remains elusive. For a few circRNAs, interesting functions have been uncovered and these have fueled speculations regarding general functions of circRNAs.

### miRNA Sponging and Binding to RBPs

The relatively well-studied circRNAs sex determining region Y (SRY) and cerebellar degeneration-related protein 1 antisense (CDR1as) have multiple binding sites for miR-138 and miR-7, respectively. Binding of specific miRNAs by these circRNAs can modulate miRNA expression levels competitively and thereby suppress their function, a process known as ‘miRNA sponging’ (Hansen et al., 2013; Memczak et al., 2013) [**Figure 1** (1)]. More recently, circHIPK3, generated from the *HIPK3* gene, was shown to mediate sponging of miR-124, thereby modulating human cell growth (Zheng et al., 2016).

Both CDR1as (also known as CiRS-7: Circular RNA Sponge for miR-7) and miR-7 have been linked to nervous system development and disease. miR-7 represses α-synuclein and miR-7 downregulation is observed in human glioblastoma (Kefas et al., 2008; Junn et al., 2009). Increased levels of miR-7 have been detected in neuropsychiatric disorders and evidence for CDR1as-mediated regulation of dendritic spine density via a miR-7-SHANK3 axis has been found (Choi et al., 2015; Zhang et al., 2015). Expression of human CDR1as in zebrafish causes impaired midbrain development. Knockdown of CDR1as leads to migration defects *in vitro*, as shown in a wound-healing assay using HEK293 cells. Whether these effects truly depend on CDR1as-miR7 interactions remains to be determined (Hansen et al., 2013; Memczak et al., 2013). Nevertheless, these data implicate a role for circRNAs in neurite growth and neuron migration [**Figure 1** (2)].

Circular RNAs might not only act as competitive RNAs for miRNAs, but could also be involved in their storage, sorting, and localization. Interestingly, CDR1as binds to Argonaute (AGO), an effector protein in the RNA-induced silencing complex (RISC). CDR1as is not cleaved by AGO upon miR-7 binding, because none of the miR-7 binding sites is complementary for more that 12 nucleotides. However, CDR1as contains a binding site for another miRNA, miR-671, which is fully complementary and can induce AGO-mediated cleavage of this circRNA (Hansen et al., 2011). This may have several co-regulatory consequences. For example, cleavage of the circRNA may result in the (local) release of binding partners (miR-7), which would indicate a role in miRNA transport and delivery [**Figure 1** (3)]. In addition, a cleaved antisense circRNA might bind its linear sense transcript, potentially resulting in RNA destabilization and silencing.

In addition to miRNAs, circRNAs bind to, sequester and transport RBPs (Hentze and Preiss, 2013; Wilusz and Sharp, 2013), which may regulate the interaction of RBPs with their RNA targets (Salzman et al., 2012) [**Figure 1** (4,5)]. Examples include circRNAs from the *muscleblind* (*mbl)* and *FOXO3* genes (Ashwal-Fluss et al., 2014; Du et al., 2016). Interestingly, alternative splicing of circRNAs may also lead to the formation of new binding sequences for specific RBPs and give rise to a number of different protein decoys. circRNAs are likely to not only bind single RBPs but actually have been proposed to act as scaffolds for the assembly of large protein complexes (Jeck and Sharpless, 2014; Lasda and Parker, 2014; Hansen et al., 2015) [**Figure 1** (6)].

Currently, it is not clear whether miRNA sponging and RBP binding are shared functions of circRNAs. Some studies demonstrate that only a few circRNAs have properties similar to the previously described miRNA sponges (Guo et al., 2014). The existence of circRNAs in species that lack RNA interference (RNAi) pathways also points toward other mechanisms. Therefore, miRNA sponging and protein binding might be exceptional properties of select circRNAs that are not shared by the majority of circRNAs.

### Transcriptional Control

As functional circRNAs have been shown to act as miRNA sponges or protein decoys in the cytoplasm, it is tempting to speculate that circRNAs primarily act as post-transcriptional regulators of gene expression (Hansen et al., 2013; Memczak et al., 2013). However, circRNAs do not only localize to the cytoplasm, but some are also present in the nucleus. circRNAs containing introns, like EIciRNAs, are retained in the nucleus, and can interfere with gene transcription, including the transcription of their parental genes. This process can be mediated by direct interaction with U1 snRNP: an EIciRNA-U1 complex can recruit RNA polymerase II (Pol II) to the promoter region of a gene, stimulating initiation of transcription [**Figure 1** (7)]. Nuclear circRNAs could therefore be specifically involved in the regulation of transcriptional activity in neurons (Zhang et al., 2013; Li et al., 2015b). For example, upregulation of *PAIP2* by circPAIP2 may affect contextual memory (Khoutorsky et al., 2013).

### Translation

Circular RNAs often contain coding exons (e.g., for protein structural domains) and carry open reading frames (Salzman et al., 2012; Enuka et al., 2015). Experimental evidence suggests that circRNAs may have translation potential [**Figure 1** (8)]. For example, synthetic exonic circRNAs can be translated *in vitro* and *in vivo* when internal ribosome entry sites (IRES) or prokaryotic binding sites are introduced (Chen and Sarnow, 1995; Perriman and Ares, 1998; Wang and Wang, 2014). If such translational products would exist endogenously, they may exert specific biological effects or interfere with protein-protein interactions. However, thus far little evidence supports the idea that circRNAs are associated with ribosomes (Shigeoka et al., 2016), so it remains unclear if and how they are translated into protein under normal conditions (Capel et al., 1993; Jeck et al., 2013; Guo et al., 2014; Granados-Riveron and Aquino-Jarquin, 2016).

### Neuronal Development and Plasticity

Several studies have demonstrated that circRNAs are specifically enriched in brain tissue. Intriguingly, circRNAs are not homogenously distributed throughout the nervous system but are differentially expressed in various brain regions and subcellular compartments, and at specific embryonic and postnatal stages (Memczak et al., 2013; Rybak-Wolf et al., 2015; Venø et al., 2015; You et al., 2015; Chen and Schuman, 2016). For example, during pig brain development several circRNAs display higher expression in the cerebellum as compared to the brainstem. It is also reported that circRNAs can be up- or downregulated in cultured neurons in response to fluctuations in neuronal activity induced by addition of the GABA_A_ receptor antagonist bicuculline (You et al., 2015). This indicates that neuronal expression of circRNA is activity-dependent [**Figure 1** (9)]. Interestingly, neuronal activity is known to affect gene expression in multiple ways, and one potential novel way may thus be by interfering with circRNA levels.

Recent studies reveal that many of the circRNAs expressed in neural tissues derive from synaptic genes such as *Dscam* and *Homer1*, or from genes with prominent roles in early neural development, e.g., genes implicated in Wnt signaling, axon guidance, and TGF-β signaling (Venø et al., 2015). A large group of circRNA transcripts, especially from axon guidance cues (e.g., from the *Eph* and *Robo* gene families) are observed to be upregulated at specific developmental time points, e.g., during synaptogenesis (Venø et al., 2015; You et al., 2015; Zhang et al., 2016). Interestingly, circRNAs from genes with synaptic functions (e.g., *Dscam* and *Homer 1*) were enriched in dendritic structures regardless of gene expression levels. Additionally they were found to be enriched in hippocampal synaptosomes, suggesting active transport of circRNAs to synapses (Rybak-Wolf et al., 2015; You et al., 2015) [**Figure 1** (10)]. In general, circRNAs were found to be highly enriched in synapses, both *in vitro* (in cell lines and primary neuronal cultures) and *in vivo* (Rybak-Wolf et al., 2015), demonstrating a heterogeneous distribution of circRNAs within neurons and implicating a role for circRNAs in neuronal development and plasticity.

Circular RNAs not only serve physiological functions in neurons, but have also been linked to brain disease. General insults to neurons, like neurotoxicity or injury, induce a stress response that involves changes in gene expression, splicing, and altered expression of ncRNAs, including circRNAs. Accumulating evidence suggests that circRNAs may be involved in a wide range of neuronal stress responses and that their aberrant expression or function may contribute to the development, progression and/or severity of various neurological disorders. For an overview of putative roles of circRNAs in neurological diseases, we refer to other recent reviews (Shao and Chen, 2016).

## Outlook

The complex anatomical organization of neurons requires precise spatiotemporal regulation of gene expression. Many RNAs and proteins are transported into neuronal extensions, axons and dendrites, where they are locally translated or activated upon stimulation by specific intra- or extracellular cues. It is plausible that some circRNAs act as local regulators of gene expression at distal neuronal sites, such as growth cones and synapses, and contribute to aspects of neural development. For example, circRNAs generated from the axon guidance receptor gene *DCC* contain a substantial amount of open reading frames encoding for ligand-binding domains. It is tempting to speculate that, if generated, these circRNA-derived protein fragments may interfere with DCC ligand binding. However, much more functional circRNA studies are needed to dissect their roles and mechanism-of-action during neural development and plasticity.

In addition to a void of data on the functional roles of circRNAs, the detection and identification of circRNAs is difficult due to the presence of sequencing errors, alignment errors, and *in vitro* artifacts. It is often challenging to interpret the heterogeneous results arising from the use of different bioinformatics methods or of sequencing data generated under different conditions (Chen et al., 2015; Hansen et al., 2015). Therefore, it is thought that only systems biology approaches provide rigorous insights into quantitative and qualitative regulation of and through circRNAs. For example, approaches that take into account RNA networks and *trans*-acting factors (transcription and splicing factors, RBPs, other interactors), and correlate expression patterns to phenotypic data are needed to answer many of the open questions in the field of neuronal circRNA research (**Box 1**).

**BOX 1. Outstanding questions.**

• Which are the functional roles of neuronal circRNAs?
• Are miRNA sponging and RBP binding general properties of circRNAs?
• Which *cis* and *trans* regulatory mechanisms control the biogenesis of neuronal circRNAs?
• How is the spatiotemporal and activity-dependent expression of circRNAs controlled?
• How are circRNAs transported in neurons and secreted?
• Are neuronal circRNA sequences and functions evolutionary conserved between species?
• Do circRNAs contribute to the development and progression of neurological disorders?
• Can circRNAs be exploited as tools, biomarkers and targets for therapeutic strategies?

In addition to their putative biological role in neurons, circRNAs are potentially interesting tools to study neurons and neural development because of their ability to bind other RNAs and proteins, their high stability and specific localization patterns. Although not discussed in detail in the current review, circRNAs are also viewed as targets for designing therapeutic approaches in brain disease. For example, circRNAs are closely intertwined with RNA processing (like pre-RNA splicing and RNA editing), which are affected in neurological diseases such amyotrophic lateral sclerosis (ALS) and spinal muscular atrophy (SMA) (Scotti and Swanson, 2015). The splicing of circRNAs could be modulated with antisense oligonucleotides and other small molecules (Havens et al., 2013; Jeck and Sharpless, 2014), leading to less potential disease-causing circularized products. Interestingly, circRNAs have been also shown to accumulate during aging (Westholm et al., 2014), which could link them to age-related neurodegenerative diseases. circRNA turnover might be lower in neurons due to their post-mitotic state. Apart from acting as therapeutic targets, circRNAs might serve as biomarkers for disease, given their stability and secretion in extracellular vesicles (Lu and Xu, 2016) [**Figure 1** (11)]. The biological role of this secretion is unknown but may be linked to communication between different cell types (e.g., neuron-glia) or different tissue compartments (including, for example, CSF and blood) (Li et al., 2015a; Lasda and Parker, 2016).

## Author Contributions

DvR and BV performed literature research and generated **Figure 1**. DR, BV, and RP wrote the manuscript.

## Conflict of Interest Statement

The authors declare that the research was conducted in the absence of any commercial or financial relationships that could be construed as a potential conflict of interest.
